# SIroliMus coated angioPlasty versus plain balloon angioplasty in the tREatment of dialySis acceSs dysfunctION (IMPRESSION): study protocol for a randomized controlled trial

**DOI:** 10.1186/s13063-021-05920-3

**Published:** 2021-12-20

**Authors:** Suh Chien Pang, Ru Yu Tan, Edward Choke, Jackie Ho, Kiang Hiong Tay, Apoorva Gogna, Farah G. Irani, Kun Da Zhuang, Luke Toh, Shaun Chan, Pradesh Krishnan, Kristen A. Lee, Sum Leong, Richard Lo, Ankur Patel, Bien Soo Tan, Chow Wei Too, Jasmine Chua, Ren Kwang Alvin Tng, Tjun Yip Tang, Siew Ping Chng, Tze Tec Chong, Hsien Ts’ung Tay, Hao Yun Yap, Julian Wong, Rajesh Babu Dharmaraj, Jun Jie Ng, Anil Gopinathan, Eu Kuang Loh, Shao Jin Ong, Gary Yoong, Jia Sheng Tay, Kay Yuan Chong, Chieh Suai Tan

**Affiliations:** 1 Department of Renal Medicine, Singapore General Hospital, Academia, Level 3, 20 College Road, Singapore, 169856 Singapore; 2 Vascular and Endovascular Service, Sengkang General Hospital, 110 Sengkang East Way, Singapore, 544886 Singapore; 3 Department of Cardiac, Thoracic & Vascular Surgery, National University Hospital, NUHS Tower Block, Level 9, 1E Kent Ridge Road, Singapore, 119228 Singapore; 4 Department of Vascular and Interventional Radiology, Singapore General Hospital, Outram Road, Singapore, 169608 Singapore; 5 Department of Vascular Surgery, Singapore General Hospital, Academia, Level 5, 20 College Road, Singapore, 169856 Singapore; 6 Department of Diagnostic Imaging, National University Health System, 5 Lower Kent Ridge Road, Singapore, 119074 Singapore; 7 Division of Medicine, Singapore General Hospital, SingHealth Tower, Level 5, Singapore, Singapore

**Keywords:** Drug-coated balloon, Sirolimus, Dialysis access dysfunction, Hemodialysis

## Abstract

**Background:**

Percutaneous transluminal angioplasty is the current standard treatment for arteriovenous fistula (AVF) stenosis. The mid- and long-term patency with plain balloon angioplasty (PBA) is however far from satisfactory. While paclitaxel-coated balloon angioplasty has been shown to be superior to PBA, concern over its safety profile has recently arisen after a reported possible increased mortality risk with a meta-analysis of large lower limb studies. An angioplasty balloon with a new type of drug coating, the sirolimus-coated balloon (SCB), has been proven to improve patency in the coronary arteries. However, its effect on AV access has yet to be studied.

**Methods/design:**

This is an investigator-initiated, prospective, multicenter, double-blinded, randomized controlled clinical trial to assess the effectiveness of SCB compared to PBA in improving the patency of AVF after angioplasty. A total of 170 patients with mature AVF that requires PTA due to AVF dysfunction will be randomly assigned to treatment with a SCB or PBA at a 1:1 ratio, stratified by location of AVF and followed up for up to 1 year. The inclusion criteria include [1] adult patient aged 21 to 85 years who requires balloon angioplasty for dysfunctional arteriovenous fistula [2]; matured AVF, defined as being in use for at least 1 month prior to the angioplasty; and [3] successful angioplasty of the underlying stenosis with PBA, defined as less than 30% residual stenosis on digital subtraction angiography (DSA) and restoration of thrill in the AVF on clinical examination. The exclusion criteria include thrombosed or partially thrombosed access circuit at the time of treatment, presence of symptomatic or angiographically significant central vein stenosis that requires treatment with more than 30% residual stenosis post angioplasty, and existing stent placement within the AVF circuit. The primary endpoint of the study is access circuit primary patency at 6 months. The secondary endpoints are target lesion primary patency; access circuit-assisted primary patency; access circuit secondary patency at 3, 6, and 12 months; target lesion restenosis rate at 6 months; total number of interventions; complication rate; and cost-effectiveness. The trial is supported by Concept Medical.

**Discussion:**

This study will evaluate the clinical efficacy and safety of SCB compared to PBA in the treatment of AVF stenosis in hemodialysis patients.

**Trial registration:**

ClinicalTrials.govNCT04409912. Registered on 1 June 2020

## Background

Despite the significant advances in medical technologies, arteriovenous fistula (AVF) dysfunction remains a major morbidity for patients with end-stage renal disease (ESRD) who are dependent on hemodialysis [1]. Neointimal hyperplasia (NIH) contributed by endothelial injury from shear stress and turbulent blood flow frequently results in clinically significant stenosis, leading to diminished blood flow and thrombosis in some accesses [2, 3]. Percutaneous transluminal angioplasty (PTA) is the current therapy of choice for AVF dysfunction. However, AVF patency rates post-PTA are often hampered by endothelial denudation and further NIH caused by mechanical dilatation of stenosis with angioplasty balloons [3]. With significant scientific advances in understanding the mechanism of AVF stenosis, medical technology innovations to improve patient care and AVF outcomes have been emerging.

Drug-coated balloon (DCB) devices are one of the most exciting technologies available in recent years. By inhibiting the proliferative response to the acute trauma caused by balloon angioplasty, the DCB has shown its efficacy with improved primary patency rates of AVF post-treatment. To date, several randomized controlled trials have shown the superiority of paclitaxel-coated balloon (PCB) angioplasty over plain balloon angioplasty (PBA) in the treatment of AV access stenosis [4–8]. However, safety concerns had also arisen recently after a meta-analysis of lower limb studies reported higher 12-month mortality of 7.6% vs. 5.8% for paclitaxel-coated devices compared to uncoated devices [9]. Although similar risks have not been demonstrated in the meta-analysis on ESRD patients receiving PCB for AVF intervention [10], the safety concern on PCB invariably may limit its use, which may affect the patency rates of AV accesses of ESRD patients. On the other hand, the use of stent-graft in hemodialysis access has shown improved patency rates in arteriovenous graft (AVG) [11–14], but its role in AVF has been controversial as it may reduce the length of vessel available for cannulation.

Sirolimus-coated balloon (SCB), a new generation of DCB, has gained interest recently as an alternative to PCB. Clinical studies in coronary artery intervention using SCB for in-stent stenosis and small vessel disease have shown excellent procedural success and 6-month post-procedural patency [15, 16]. Compared to paclitaxel, sirolimus is cytostatic and has a wide therapeutic index indicating a more favorable safety profile. In addition, sirolimus has been used in ESRD patients who have received renal transplantation as immunosuppressive agents for decades at a much higher dosage than the dose on SCB. The IMPRESSION study aims to examine the effectiveness and safety of SCB angioplasty compared with PBA in the treatment of AVF stenosis.

## Trial objectives

The primary objective is to determine if the use of SCB will result in the improvement of the access circuit primary patency at 6 months when compared to PBA.

The secondary objectives are a follows:

1. To determine if the use of SCB, compared to PBA, will lead to decreased restenosis of the target lesion
2. To determine if the use of SCB, compared to PBA, will lead to improved target lesion and access circuit patency
3. To determine if the use of SCB, compared to PBA, will lead to decreased number of interventions needed to maintain patency of AVF over 1 year
4. To analyze the cost-effective of using SCB in the management of dialysis access stenoses

## Trial design

**Fig. 1:**
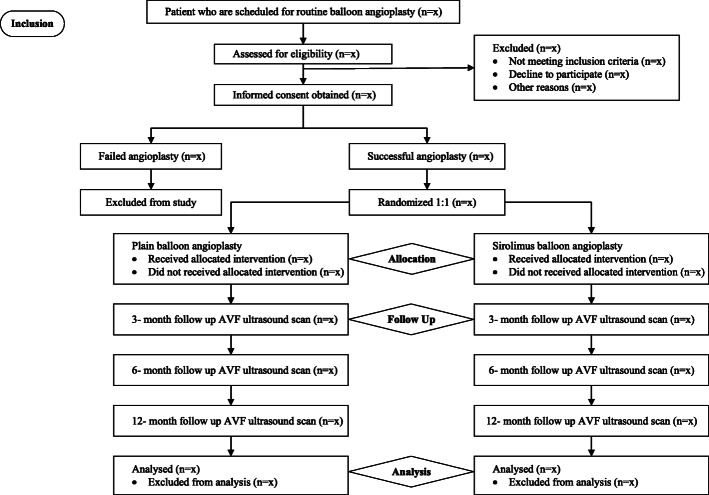
Flowchart of the study based on the Consolidated Standards for Reporting of Trials

**Fig. 2:**
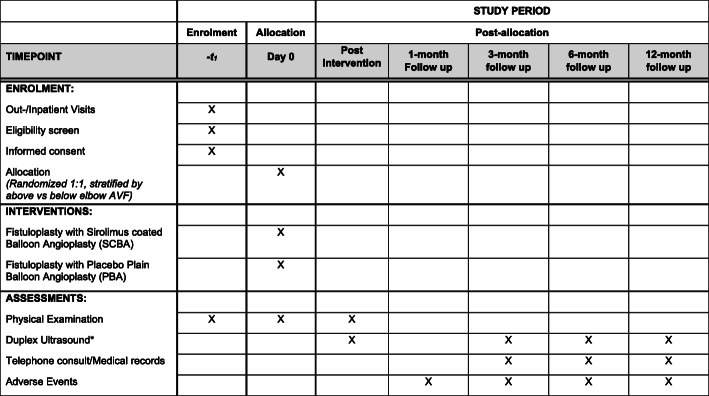
Schedule of enrollment, interventions, and assessments according to the SPIRIT 2013 Statement: Defining Standard Protocol Items for Clinical Trials.* The clinically driven fistulogram may be used in lieu of the 6-month follow-up ultrasound if it is performed within the window period of the scheduled 6-month ultrasound. The same rule applies to those patients who fall into the window period of the scheduled 12-month ultrasound

This multicenter, prospective, parallel, double-blinded, randomized controlled trial is an investigator-initiated study. The patients are randomized at a 1:1 ratio to receive either SCB (intervention arm) or PBA (placebo arm) following successful angioplasty of AVF stenosis. Randomization will be stratified by location of AVF (above vs. below elbow) to ensure an even distribution of AVF by location between both groups (Figs. 1 and 2).

## Methods: participants, interventions, and outcomes

### Participants

The study will recruit a total of 170 patients from three different hospitals in Singapore over 4 years. The potential participants are patients who are scheduled for PTA of dysfunctional AVF by their primary physician. The potential participants will be screened for eligibility according to the inclusion and exclusion criteria for the study summarized in Table 1 by the study team member. Eligible patients are offered enrollment. As one of the eligibility criteria (residual stenosis < 30%) can only be determined during the procedure, informed consent is taken from eligible participants before the procedure. The study team members will obtain written informed consent to participate in the study from all enrolled participants. Additional informed consent for the collection and the use of participant data will also be obtained. An enrollment number is given to each participant for the purpose of anonymization.

**Table 1:** Inclusion and exclusion criteria for the IMPRESSION study

Inclusion criteria	Exclusion criteria
1. Age 21 to 85 years 2. Patients who require balloon angioplasty for dysfunctional arteriovenous fistula (AVF) 3. Matured AVF, defined as being in use for at least 1 month prior to angioplasty 4. Successful angioplasty of underlying stenosis, defined as less than 30% residual stenosis on digital subtraction angiography based on visual assessment of the operator, and restoration of thrill in the AVF on examination*	1. Patient unable to provide informed consent 2. Thrombosed or partially thrombosed AVF 3. Presence of symptomatic or angiographically significant central vein stenosis who require treatment, with more than 30% residual stenosis post angioplasty 4. Patients who had underwent stent placement within the AVF circuit 5. Patient who are currently enrolled in other drug eluting balloon trials 6. Sepsis or active infection 7. Recent intracranial bleed or gastrointestinal bleed within the past 12 months 8. Allergy to iodinated contrast media, heparin, or sirolimus 9. Pregnancy

*A prominent pulsation felt on palpation of AVF suggesting elevated intra-access pressure caused by an outflow stenosis. This is often accompanied by weak or absence of thrill if the outflow stenosis is severe. Pulsation should disappear or reduced with restoration of thrill in the AVF if angioplasty of the underlying stenosis is successful

### Interventions

After enrollment, the patient will undergo PTA procedures in the study sites’ interventional suites equipped with a fluoroscopy machine with the ability to perform digital subtraction angiography and post-processing software for quantitative vascular analysis. Fistulograms of the entire dialysis circuit from the feeding artery, arteriovenous anastomosis, to the central veins will be performed. The target lesions will be treated in the standard fashion with PBA. When there is more than one stenosis, all the lesions will be labeled and treated accordingly. Lesions are considered separate if they are separated by a gap of at least 2 cm. The operator will angioplasty each lesion with a PBA sizing that is identical to the adjacent reference vessel. Inflation time will be at least 1 min per inflation. If there is a significant residual stenosis after the initial angioplasty, repeat angioplasty with a larger diameter, high-pressure, or cutting balloon may be used at the operator’s discretion. In the stenotic segment adjacent to an aneurysmal segment, where the percentage of stenosis is difficult to determine, vessel diameter must reach at least 6 mm to be considered for inclusion.

### Randomization procedures

Once all the target lesions are adequately treated, as visually assessed by the operator to have less than 30% residual stenosis, the patients will be randomized, using a secure independent web-based randomization program developed by the Singapore Clinical Research Institute (SCRI). Randomization will be performed for 170 subjects (85 subjects per arm) with the three participating centers and location of AVF (above or below elbow) as stratification factors. Randomization is based on the permuted block randomization with the above stratification factors. Each coordinator has a unique user access provided by the SCRI upon the authorization of the study principal investigator. In the same procedure room, the study coordinator will log into the website (https://rand.scri.edu.sg), enter the participant’s subject number, initials, date of birth, and location of AVF. Once randomized, the program will automatically generate a randomization number. The coordinator then refers to the treatment allocation list to determine which arm the patient has been randomized to and inform the operator.

#### Trial intervention

Based on randomization, all lesions will be treated with either study balloon “A” or “B.” The diameter of the study balloon chosen should be close to the size of the adjacent healthy segment of the blood vessel, and balloon length selection will be at the operator’s discretion. The study balloons are inflated to the appropriate pressure, not exceeding the rated burst pressure, for at least 2 min. Completion venograms of all the target lesions are performed following treatment with study balloons.

#### Study devices

The study balloons (sirolimus coated and placebo balloon) used in both arms are custom-made by the same manufacturer (Concept Medical Research Private Limited, India) and have the same profile, inflation pressure, and identical packaging. In addition, the balloons are labeled as “A” and “B” to maintain the blinding. All the balloons are of the 0.035" platform and available in diameters of 5, 6, 7, 8, and 12 mm; lengths of 60, 80, and 100 mm; and shaft length of 45 or 90 cm. The dosing of sirolimus on SCB is 1.25 μg/mm^2^.

### Blinding

The assigned operator performing the procedure will be different from the study member who performed the treatment allocation. The participants, referring physicians, investigators assessing post-intervention outcomes, dialysis center staff, investigators performing the follow-up, the operator performing the PTA, and the data analysis team will be blinded to the treatment allocation. During the procedure, the participant will not know which treatment they will be receiving as they will not be able to see the procedure with their views completely blocked from the operative field by sterile drapes. The operator will be instructed of which balloons (“A” or “B”) to use, but they will not know whether these balloons are sirolimus-coated balloons or the placebo. Only designated study coordinators handling the investigational product will know the treatment allocation as it is not feasible for them to administer the trial blinded.

### Unblinding procedure

Unblinding will occur at the end of the study (the 12-month follow-up of the last recruited participant). At this stage, all blinded research data would have been collected, and unblinding will allow the data analyses to occur. In case of an emergency requiring information on treatment allocation for patient management, the principal investigator will break the blind for the patient.

### Screen failure and study withdrawal

Patients with resistant stenosis defined as residual stenosis > 30% that cannot be successfully treated with balloon angioplasty, have partial thrombosis of the AVF, or require stent deployment will not be eligible for randomization. Such cases are considered screen failures and will be replaced. These participants will still receive the standard care with PBA.

The participants are free to withdraw their consent and discontinue participation at any time during the trial without prejudice to their medical care. Participants who choose to withdraw will not be replaced. The data that has been collected until the time of withdrawal will be kept and analyzed to enable a comprehensive evaluation and maintain the scientific validity of this study. These participants will be asked for permission to continue clinic follow-up for the assessment of safety outcomes. Any adverse events will be monitored and treated till resolution. As ESRD patients have a risk of sudden death, if any study participant passes away before the time point of primary outcome measure at 6 months, they will be considered as dropout and replaced.

### Post-procedure care, assessment, and follow-up

Post-procedure, no antiplatelet or anticoagulation will be prescribed as part of the study. The participant who is already on antiplatelet and/or anticoagulation will be resumed. Participants will return to their respective community dialysis for routine dialysis and have the AVF monitored by using a combination of dialysis parameters and transonic flow measurements. Any abnormalities related to these parameters will be referred to the study site for further assessment.

#### Immediate assessment

As ultrasound is used as an imaging tool to monitor the AVF post-intervention for the study, ultrasound assessment of the AVFs will be performed by a trained operator to document the diameter of the vessels within 24 h post-PTA. Volume flow rates at the mid-brachial artery and venous outflow will be recorded. The minimum diameter of each target lesion will be recorded.

#### Three-, 6-, and 12-month assessment

All participants will be followed up for up to 1 year. The window periods for the post-PTA visits are 3 months ± 1 week, 6 months ± 4 weeks, and 12 months ± 4 weeks. During the follow-up period, ultrasound assessment will be performed in each study site’s vascular study unit. A reminder will be given to participants by the study coordinator to ensure adherence to follow-up ultrasound. The Duplex ultrasonography assessment includes patency of the AVF, volume flow rates at the mid-brachial artery and venous outflow, minimum diameter of each target lesion, and any new stenosis within the AVF circuit. For patients who may have undergone or are planning for fistulograms, the fistulograms may be used instead of the scheduled ultrasound. A study team member who is not involved in the index procedure will be responsible for reviewing the patient, ultrasound images or reports, and hemodialysis charts during each follow-up and will determine the plans for repeat intervention when clinically indicated.

#### Contingency plan

In situations where patient to hospital visits is limited to essential visits only, the patient will not be able to return for follow-up ultrasound scans. The study team will review the patient’s medical record and dialysis records from the patient’s dialysis centers and conduct telephone consult with the patient to collect data in place of the ultrasound scan.

#### Repeat intervention on AVF

Repeat intervention will be performed when a decrease in access flow is associated with clinically significant lesions as recommended by the National Kidney Foundation’s Kidney Disease Outcomes Quality Initiative (KDOQI) clinical practice guideline for vascular access: 2019 update (Table 2) [17]. Patients who require repeat intervention on the AVF are considered to have reached the primary endpoint.

**Table 2:** Indications for reintervention of the AVF

1. Thrombosed or partially thrombosed AVF	
2. Ipsilateral extremity edema	
3. Alteration in pulse, thrill, or bruit	
4. Clinical features of inflow stenosis: lack of pulse augmentation	
5. Clinical features of outflow stenosis: failure of fistula to collapse when the arm is elevated	
6. Excessive collapse of venous segment upon arm elevation	
7. New difficulty in cannulation	
8. Aspiration of clots	
9. Inability to achieve the target dialysis blood flow	
10. Prolonged bleeding for 3 consecutive dialysis sessions	
11. Unexplained (> 0.2 units) decreased in delivered Kt/V on a constant dialysis prescription	

#### Central laboratory assessment

The index procedural fistulograms and all follow-up ultrasound images will be sent to a central laboratory for review by a group of independent assessors. The independent assessors will use quantitative vascular analysis software (Syngo, *Siemens* Healthcare, Erlangen, Germany) as an adjunct to evaluate the lesions from fluoroscopy images in the picture archiving and communication system (PACS). The central laboratory’s interpretation of all angiograms will be used for the data analyses.

## Outcomes

In this study, standard definitions for patency outcomes and major and minor complication rates according to the Society of Interventional Radiologist (SIR) guidelines are used [18].

### Primary endpoints

The efficacy endpoint is access circuit primary patency rates at 6 months.

The safety endpoint is complication rates at 1, 3, 6, and 12 months according to the Society of Interventional Radiology (SIR) definitions of minor or major complications [18].

### Secondary endpoints

The following are the secondary endpoints:

1. Time taken to the next intervention
2. Target lesion percent stenosis at 6 and 12 months with ultrasound
3. Target lesion restenosis rate at 6 months
4. Number of repeat interventions to target lesion at 6 and 12 months
5. Number of repeat interventions to maintain access circuit at 6 and 12 months (including interventions to the treated lesion)
6. Target lesion revascularization free interval
7. De novo stenosis detected on ultrasound scan at 3, 6, and 12 months
8. Post-intervention target lesion patency at 3, 6, and 12 months
9. Post-intervention primary patency at 3, 6, and 12 months
10. Post-intervention assisted primary patency at 3, 6, and 12 months
11. Post-intervention secondary patency at 3, 6, and 12 months

Access circuit primary patency rates are defined as the percentage of patients whose AVF remains patent at 6 months after the index PTA. A major complication is defined as requiring therapy, minor hospitalization < 40 h, requiring major therapy, unplanned increase in the level of care, prolonged hospitalization (> 48 h), leading to permanent adverse sequelae, or death. A minor complication requires no therapy with no consequences or requires nominal therapy with no consequences, including overnight admission for observation only [18].

Time taken to the next intervention is defined as the number of months from index angioplasty to the subsequent intervention or till study completion at 12 months. Target lesion percent stenosis is defined as percent stenosis relative to adjacent reference vessel ([1 − (minimum lesion diameter/reference vessel diameter)] × 100) on 6- and 12-month follow-up ultrasound. Restenosis rate is defined as the incidence of more than 50% diameter narrowing of the target lesion compared to adjacent vessel segment at a 6-month follow-up ultrasound scan. Target lesion re-intervention-free interval is defined as the interval from index angioplasty to repeat clinically driven target lesion intervention, anytime within the 12-month study period.

Post-intervention primary patency is defined as the percentage of patients whose AVF remains patent and does not require any further interventions [18], while post-intervention target lesion patency is measured as the percentage of patients whose AVF remains patent and does not require any additional interventions at 3, 6, and 12 months after the index angioplasty. These outcomes are determined by ultrasound imaging or angiogram or clinical examination. The decision for reintervention based on clinical examination findings includes loss of thrill, pulsatile flow, or swollen arm. Post-intervention assisted primary patency is defined as the percentage of patients whose AVF requires additional interventions to remain patent, and post-intervention secondary patency is measured as the percentage of patients whose AVF have thrombosed and require additional procedure to restore flow at 3, 6, and 12 months after the index angioplasty [18]. These outcomes are determined by clinical history during the study period.

## Statistical analysis

### Sample size calculation

We assume that the SCB will have a similar effectiveness as the PCB based on a previous meta-analysis of published RCT, the 6-month target lesion primary patency 73.7% with PCB vs. 55.2% with plain balloon angioplasty. Considering a dropout rate of 10%, a sample size of 170 patients randomized into a 1:1 ratio will have 80% power to detect a difference between the two groups at 6 months.

### Primary outcome

Both the primary efficacy outcome and primary safety outcome will be analyzed using an intention-to-treat (ITT) analysis set which includes all randomized subjects. The ITT subjects will be analyzed according to their randomized group assignment irrespective of the treatment delivered and subject follow-up time, and all events post-randomization will be counted toward study endpoints. The count and percentage of subjects with each outcome will be presented by treatment. The percentage of the efficacy endpoint will be based on the subjects who had a non-patency event (i.e., CD-TLR or access circuit thrombosis) within 210 days post-procedure or had no non-patency event but followed up for at least 150 days. The efficacy endpoint will be compared between treatments using the *Z*-test (*Z*-test approximation to a binomial distribution) as the primary analysis method. The percentage of the primary safety endpoint will be based on subjects who had access circuit-related SAE within 30 days post-procedure or had no access circuit-related SAEs but were followed up for at least 23 days. Non-inferiority on the safety endpoint will be tested using the Farrington-Manning method. The differences between treatments and the corresponding 95% confidence interval (CI) will be calculated. To control the overall type I error, the following sequential analysis approach will be taken:

- Primary efficacy superiority; if significant at one-sided alpha = 0.025
- Primary safety non-inferiority; if significant at one-sided alpha = 0.025
- Then proceed to key secondary endpoints

The study will be deemed a success if both the superiority of efficacy and non-inferiority of safety are demonstrated. Additional analysis of the primary endpoint using the time to the events will be evaluated according to the Kaplan-Meier method, and the log-rank tests will be applied to compare the survival curves over time between the treatments for each primary endpoint respectively.

### Secondary outcome

Descriptive statistics for the secondary endpoints will be provided. Unless otherwise specified, for categorical variables, the count and percentage of subjects with each outcome will be presented. They will be evaluated by using the chi-square tests or Fisher’s exact tests depending on the event counts. Continuous variables will be compared with *t*-tests. The differences between treatments together with the corresponding 95% confidence interval will be calculated. Additional time to event survival analysis will be employed when applicable. Secondary endpoints will be analyzed using ITT analysis set, per-protocol analysis set, and as-treated analysis set when applicable. The key secondary endpoints will be compared between the treatments sequentially by using ITT analysis set in a superiority manner if the two primary endpoint tests pass, each at a one-sided significance level of 0.025.

As-treated analysis set includes randomized subjects who received a SCB or PBA. The as-treated subjects will be analyzed according to the device subjects received. If the as-treated analysis set is different from the ITT analysis set, the primary and secondary endpoints will be analyzed on the as-treated analysis set to assess the sensitivity. The per-protocol analysis set includes subjects who have (a) received the randomized treatment as assigned without provisional stenting or other potential bailout procedure, (b) no pre-specified inclusion/exclusion violation(s), and (c) available endpoint data post-index procedure. The per-protocol analysis set will be applied to primary and key secondary endpoint analyses.

### Safety analysis

All adverse events (AEs) post-informed consent will be collected and presented in a listing. The AEs started during or post index procedure through the end of study will be tabulated. The AEs, significant adverse events (SAEs), and AEs leading to death will be summarized by treatment, by system organ class, and by time. The relationship of AEs to procedure, device, and therapy will also be summarized. The Fisher exact test will be used to test the treatment differences.

## Ethics and regulatory approvals

The trial will be conducted in compliance with the principles of the Declaration of Helsinki (1996). This study protocol and all its related documents have been approved by the local Institutional Review Board (reference: 2019/2896). Informed consent will be obtained from all subjects participating in the study. In the event the patient is unable to write, informed consent can be given via a thumbprint or orally in the presence of at least one witness in accordance with the Medical Research Involving Human Subjects Act (article 6, subsection 2, altered WMO).

## Data handling and auditing

Hardcopy source documents will be used to collect the required data. The contained information will then be entered into the Electronic Research Data Capture (REDCap) system by the study coordinator. The hardcopy source document and a list containing the links between the enrollment numbers of each participant to their identities will be kept under lock and key cabinets. Online database/electronic case report forms will be password-protected. Only the principal investigator, designated co-investigators, and study coordinators will have access to the research data. Access to the hardcopy data will be controlled. Access to the electronic database/case report forms will be password-protected and login-recorded. The images from the angiogram and intervention and ultrasound scans are recorded into the electronic picture archiving and communication system (PACS) of a password-protected computer as per routine clinical practice in the study site. These records will be anonymized and saved in a hard disk for review by independent assessors.

### Data monitoring committee (DMC)

The data and safety monitoring will be performed by the principal investigator and a team of co-investigators. The principal investigator and study coordinators will be responsible for the dissemination of data and safety information to the study sites. This will be communicated via face-to-face meetings and emails using secure institution password-protected electronic communications.

## Assessment of safety

All adverse events are recorded in the case report forms. The principal investigator and co-investigator will monitor safety data by reviewing the case report forms. All serious adverse events will be notified by the principal investigator to the CIRB within the stipulated timeframe. Follow-up information will be actively sought and submitted as it becomes available.

## Discussions

We present the protocol of a multi-center RCT to evaluate the efficacy of SCB in improving patency rates of AVFs from a reduction in restenosis and reintervention rates. SCBs have been used successfully in hemodialysis accesses in small, non-randomized studies. Tan et al. reported a 3- and 6-month primary patency of 76% and 65%, respectively, with the application of SCB at the graft vein junction after successful thrombectomy of AVG [19], while Tang et al. reported a 3- and 6-month target lesion primary patency of 97.9% and 82.9%%, respectively, following treatment with SCB for dysfunctional AVF [20]. Although both studies suggested that SCB may be safe and efficacious for the treatment of hemodialysis access dysfunction, one cannot draw a conclusion from these small, pilot studies without control groups.

To the author’s knowledge, this is the first RCT comparing SCB use versus PBA in dysfunctional AVF. Double blinding is used in the study to minimize potential bias. To reduce the confounding effects from non-maturing AVF that may have a different pathogenesis, the study includes only mature AVF which has been in use for at least 1 month. In addition, randomization is stratified by location of AVF (above vs. below elbow) to minimize the heterogeneity between both groups, as above-elbow AVFs generally have larger vessel sizes and may have better outcomes than below-elbow AVFs.

Access circuit primary patency instead of target lesion primary patency is chosen as the primary endpoint because the study is designed to treat all stenosis in the AVF circuits. Treatment of only the single most severe stenosis in the access circuit with DCB could dilute the effect of DCB in prolonging access circuit primary patency. Furthermore, it is the access circuit patency that is most meaningful to the patients with numerous economical and psychosocial benefits from a lesser need for reintervention. In accordance with the KDOQI Clinical Practice Guideline for Vascular Access 2019, reintervention of AVF is based on clinical indications as summarized in Table 2 [17]. A recruited patient who has concurrent asymptomatic central vein stenosis will not receive intervention for the central vein stenosis as previous evidence showed a lack of benefit in treatment in this group of patients [21].

PBA may exacerbate NIH resulting in restenosis following interventions. DCB has been shown to blunt the acceleration of NIH and has the potential benefit to reduce the re-intervention rate. As the primary role of DCB is to allow delivery of drug to the vessel wall and penetrates the vessel tissue layers more readily, their lower pressure, semi-compliant balloon profile might not be adequate to treat resistant fibrotic stenosis if used alone. Previous DCB studies used high-pressure balloon if a plain balloon failed to treat the target lesion adequately, and they reported a high anatomic success rate of 85 to 100% [4, 5, 7, 8, 22]. In addition, the KDOQI Clinical Practice Guideline for Vascular Access 2019 recommends the use of high-pressure balloon angioplasty as the primary treatment of both clinically and angiographically significant AVF lesion [17]. The use of high-pressure or cutting balloons is permitted in our study and is likely to increase the chance of achieving this goal—adequate vessel preparation. Important to note that while these DCB studies were designed differently, they showed a similar primary efficacy endpoint of target lesion at 6 months with DCB after adequate vessel preparation. We believe adequate pre-treatment is likely to result in better vessel wall and balloon apposition which enhances drug penetration into the vessel wall, allowing maximal pharmaceutical effects to inhibit neointimal hyperplasia. Hence, to allow efficacy assessment of SCB in prolonging access circuit primary patency of AVF, adequate vessel preparation of all lesions is a pre-requisite before entering randomization in our study.

In this study, we use ultrasound as a tool to assess the AVF following angioplasty; this is to examine and document the degree of post-procedural elastic recoil within 24 h. This will help determine whether the stenosis seen in subsequent follow-up ultrasound is part of the NIH process or recoil post-angioplasty. The rate of late lumen loss may also be compared accurately between the two groups. Ultrasound assessment also allows a detailed evaluation of any potential vascular injury/dissection during the index procedure and monitor for recovery. Systemic use of sirolimus has been associated with an increased risk of impaired wound healing [23]. Locally delivered sirolimus was thought to be responsible for impaired re-endothelialization and lead to aneurysm formation following sirolimus-eluting stent placement reported in the coronary artery interventions [24, 25]. During the follow-up period in this study, ultrasound will allow any potential vascular malformations to be detected, recorded, and followed up longitudinally. We anticipate that the result of this trial will provide additional insight into the effort to improve patency outcomes in AVF for ESRD patients.

## Trial status

Recruitment started on January 11, 2021. The projected timeline for recruitment and follow-up is expected to finish by May 2024.

## Data Availability

The datasets generated and/or analyzed during the current study are not publicly available due to confidentiality of the data but are available from the corresponding author on reasonable request.

## Abbreviations

AVF: Arteriovenous fistula
NIH: Neointimal hyperplasia
DCB: Drug-coated balloon
SCB: Sirolimus-coated balloon
AV: Arteriovenous
PCB: Paclitaxel-coated balloon
ESRD: End-stage renal disease
DSA: Digital subtraction angiography
PTA: Percutaneous transluminal angioplasty
